# Screening for potential prophylactics targeting sporozoite motility through the skin

**DOI:** 10.1186/s12936-018-2469-0

**Published:** 2018-08-31

**Authors:** Ross G. Douglas, Miriam Reinig, Matthew Neale, Friedrich Frischknecht

**Affiliations:** Integrative Parasitology, Center for Infectious Diseases, Heidelberg University Medical School, Im Neuenheimer Feld 324, 69120 Heidelberg, Germany

**Keywords:** *Plasmodium*, Sporozoite, Gliding motility, MMV Malaria Box, Gramicidin, Monensin

## Abstract

**Background:**

Anti-malarial compounds have not yet been identified that target the first obligatory step of infection in humans: the migration of *Plasmodium* sporozoites in the host dermis. This movement is essential to find and invade a blood vessel in order to be passively transported to the liver. Here, an imaging screening pipeline was established to screen for compounds capable of inhibiting extracellular sporozoites.

**Methods:**

Sporozoites expressing the green fluorescent protein were isolated from infected *Anopheles* mosquitoes, incubated with compounds from two libraries (MMV Malaria Box and a FDA-approved library) and imaged. Effects on in vitro motility or morphology were scored. In vivo efficacy of a candidate drug was investigated by treating mice ears with a gel prior to infectious mosquito bites. Motility was analysed by in vivo imaging and the progress of infection was monitored by daily blood smears.

**Results:**

Several compounds had a pronounced effect on in vitro sporozoite gliding or morphology. Notably, monensin sodium potently affected sporozoite movement while gramicidin S resulted in rounding up of sporozoites. However, pre-treatment of mice with a topical gel containing gramicidin did not reduce sporozoite motility and infection.

**Conclusions:**

This approach shows that it is possible to screen libraries for inhibitors of sporozoite motility and highlighted the paucity of compounds in currently available libraries that inhibit this initial step of a malaria infection. Screening of diverse libraries is suggested to identify more compounds that could serve as leads in developing ‘skin-based’ malaria prophylactics. Further, strategies need to be developed that will allow compounds to effectively penetrate the dermis and thereby prevent exit of sporozoites from the skin.

**Electronic supplementary material:**

The online version of this article (10.1186/s12936-018-2469-0) contains supplementary material, which is available to authorized users.

## Background

The increasing emergence of resistance to front-line anti-malarial drug artemisinin emphasizes the need for identification and development of novel drug candidates [1–3]. To reduce the occurrence of drug resistance, the malaria parasite *Plasmodium* should be blocked at multiple stages of the life cycle [3–7]. In line with this, many studies have attempted to screen for compounds that are potent inhibitors of liver stage development, blood stage growth, gametocyte integrity, or transmission into the mosquito (or a combined potency of all of these) [8–27]. While often overlooked, the sporozoite stage of the life cycle presents a possible opportunity for prophylaxis [28–32]. Sporozoites form in oocysts within the mosquito vector and need their motility first to be released into the haemocoel of the insect [33], where they passively drift before actively invading salivary glands [34–36]. During mosquito probing for a blood meal, sporozoites flow out with the saliva and are deposited in the skin of the mammalian host [30, 37–40]. Sporozoites, powered by an actomyosin system, move rapidly through the dermis using a form of locomotion referred to as gliding motility [30, 41, 42]. Sporozoites then associate with blood vessels and enter the blood stream whereby they passively drift before invading hepatocytes [29, 30, 43–45].

Sporozoites are a viable target for malaria prophylaxis for several reasons. Firstly, sporozoite deposition into the skin presents a population bottleneck. Approximately 1–100 sporozoites are introduced into the skin during probing and thus only a small number of parasites need to be inhibited and/or cleared by the immune system [30, 38, 46]. Secondly, the skin step is the longest extracellular stage of the life cycle in the human host (estimated to be more than 10 min) [37] and thus, due to this long exposure outside of a host cell, might be possibly more vulnerable to appropriate drugs or immune responses than merozoites. Stalling sporozoites in the skin could allow for sufficient time for the phagocytic cells of the immune system to clear them [47]. Indeed, inhibiting sporozoite migration can be achieved by antibodies targeting the circumsporozoite protein CSP [42, 48, 49]. Thirdly, sporozoites might possibly be targeted by compounds directly applied to the skin, perhaps administered in the form of a daily body lotion or soap, thus avoiding the difficult pharmacological parameters of toxicity and bioavailability that many orally administered candidates encounter. Lastly, inhibitors of sporozoite motility could display broader inhibition of other stages and thus might also inhibit the active invasion of merozoites (needed for red blood cell invasion) and motility of midgut penetrating ookinetes.

To date, there have been no compound library screens performed on whole sporozoites to identify direct inhibitors of extracellular sporozoite motility. Here, the results of screens using two available drug libraries against motile sporozoites are presented. Using this approach, three compounds from the MMV Malaria Box (out of six initial hits) and antimicrobial ionophores monensin and gramicidin were identified as possible lead candidates for the potential use in malaria skin phase prophylaxis should an appropriate delivery method become available. These data also show that only a few compounds show inhibitory effects, suggesting that compounds identified from screens against a multiplying parasite might not be suited for repurposing to inhibit motile extracellular stages.

## Methods

### Compound libraries

Two compound libraries were tested for effects on isolated sporozoites: the MMV Malaria Box and a shortlisted version of a library containing FDA-approved drugs [8]. The Malaria Box is a library of approximately 400 compounds that were initially identified as hits of *Plasmodium falciparum* asexual blood stage development [10] and later screened for effects at other life cycle stages as well as different pathogens [9]. The FDA-approved drug library was made up of 1037 drugs that have received approval for human or animal use in the treatment of a spectrum of diseases. This library was chosen as any hits identified from this screen would possess desired drug-like properties, thereby accelerating the discovery to application process and thus save time, money and licensing complications. A screen of the entire library against *Plasmodium berghei* liver stage invasion and/or development has already been performed [8]. Ninety-seven relevant hits from this screen (which could be targeting motility, invasion and/or intracellular development) were acquired as a potential drug short-list and assessed for direct effects on sporozoites.

### In vitro screening assay for sporozoite motility inhibition

Rodents infected with *P. berghei* (NK65 strain expressing GFP under the CSP promoter) were anesthetized, and starved mosquitoes allowed to feed. Salivary glands of infected mosquitoes (days 17–24 post-infection) were isolated by dissection, parasites placed into RPMI-1640 P/S buffer (supplemented with 50,000 units l^−1^ penicillin and 50 mg l^−1^ streptomycin), released by mechanical crushing and briefly centrifuged. Sporozoites were resuspended in activation medium (RPMI-1640 P/S supplemented with 6% bovine serum albumin) and aliquoted into a 384-well plate to a final amount of approximately 2000 sporozoites per well. An equal volume of inhibitor (in RPMI-1640 P/S) was added promptly to the sporozoites to give a final concentration of either 1 µM or 10 µM and mixed by gentle pipetting. Some compounds of the Malaria Box were excluded due to high background fluorescence and thus affected the visualisation of sporozoites: MMV006309 and MMV009127. The sporozoite-inhibitor mixture was centrifuged for 3 min at 1000 rpm to maximise sporozoite numbers adhering to the glass, incubated for 30 min and each well imaged at 1 Hz for 30 s. To identify a maximum number of compounds, and due to the large number of compounds screened, the limitations of the number of sporozoites that one needs for screening and the nature of the assay itself (live imaging on parasites that only move for a short time), all initial hit identification in the pilot screen was done with a single assay per compound. All compounds from both libraries were first assessed with automated tracking software ToAST [50] and subsequently visually for changes in motility (by maximum intensity z-projections) and sporozoite morphology. Compounds that showed potent inhibition in the pilot screen were further assessed for inhibition reproducibility with at least two additional biological replicates. Sporozoites were classed as moving if they moved more than 1 parasite length during the 30-s acquisition. The percentage residual motile population was then calculated and compared to uninhibited controls (buffer solution containing an equivalent amount of DMSO). Compounds displaying > 75% inhibition at these conditions were considered for in vivo characterization.

### In vivo validation of Tyrosur^®^ gel

To fully characterize the in vivo efficacy of one of the hits, a rodent model of infection was employed. All animal experiments were performed according to FELASA B and GV-SOLAS standard guidelines. Animal experiments were approved by the German authorities (Regierungspräsidium Karlsruhe, Germany). Approximately 100 mg of Tyrosur^®^ gel formulation (Engelhard Arzneimittel) was applied to the ear 4 h before the bite experiment. Mice were then anesthetized with a ketamine/xylazine mixture (87.5 mg kg ketamine^−1^, 12.5 mg kg^−1^ xylazine, administered intraperitoneally) and infected mosquitoes allowed to bite on a single mouse ear for 10–20 min. For in vivo imaging on the ears of living mice, mice were anesthetized as above and the hair removed by treatment with hair removal cream (Veet) for 5 min 24 h prior to mosquito bite. The next day, mice were again anesthetized and infected mosquitoes allowed to bite the treated ear. Upon observation of a mosquito bite, the anesthetized mouse was immediately moved onto a heated chamber wide field microscope (Zeiss Axiovert TM200), the bite site identified and sporozoites imaged at 1 frame every 3 s for 5 min as described previously [51]. To monitor infection progression post-mosquito bite, mice were given standard drinking water and monitored daily from day 3 after biting by Giemsa staining to measure the prepatent period and subsequent blood stage growth and compared to control mice, which were treated by a control cream (Vaseline). Mann–Whitney statistical tests were conducted for non-parametric datasets.

## Results

### MMV Malaria Box screen identifies potent inhibitors of sporozoite motility

**Fig. 1:**
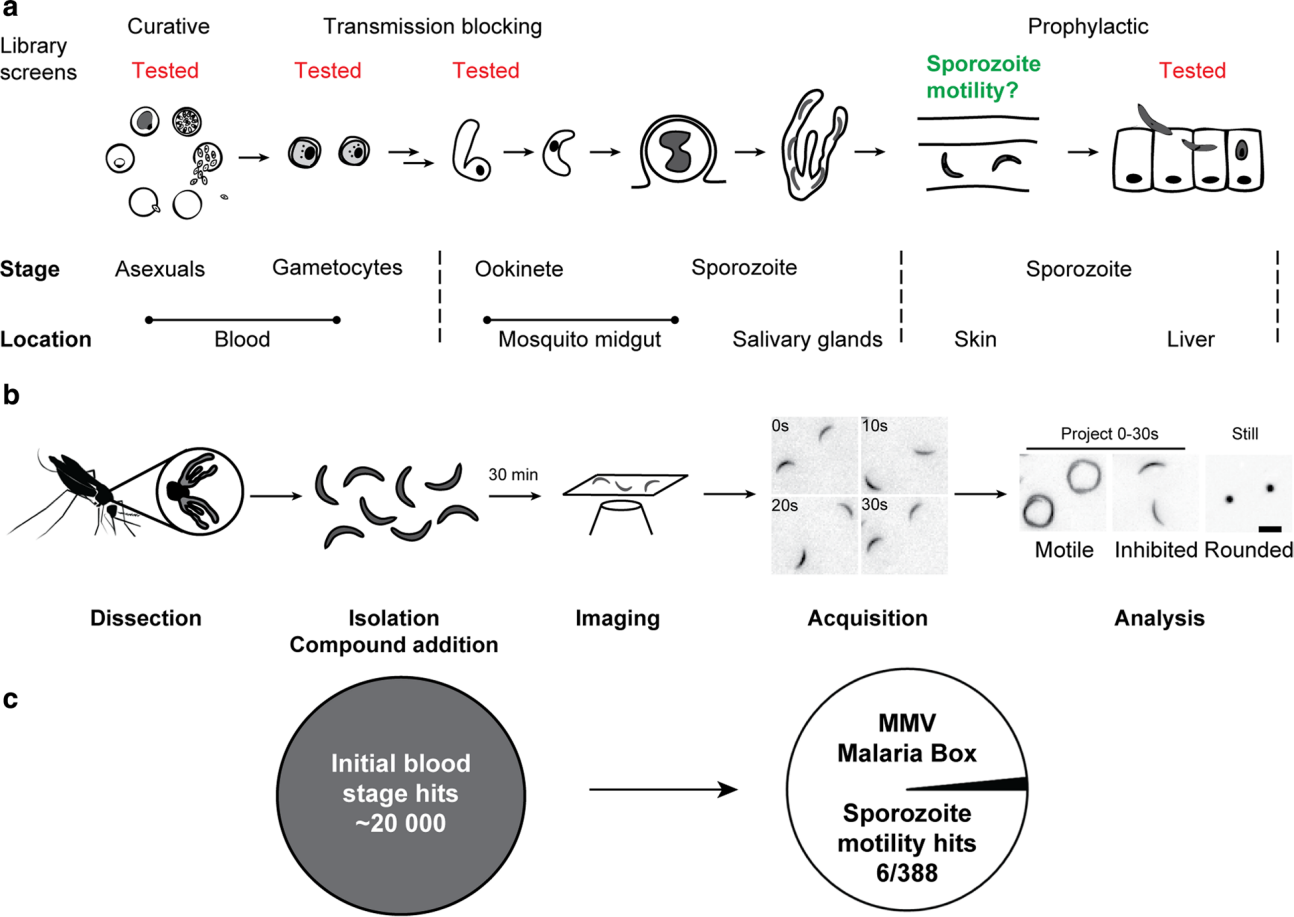
Overview of previous and current screening strategies. **a** Previously, researchers have screened for compounds active at multiple stages (indicated in red text) yet there are no studies directly attempting to stop skin phase sporozoites (green text). **b** Summary of the current screening approach. *Plasmodium berghei* sporozoites are isolated from freshly dissected *Anopheles stephensi* mosquitoes and incubated with compounds for 30 min. Sporozoites are imaged and the effects on motility and morphology noted. Scale bar: 10 μm. **c** The Malaria Box is a condensed hit library from a large set of blood stage positive hits. The motility screen identified 6 compounds that displayed noticeable inhibition of extracellular sporozoites

**Fig. 2:**
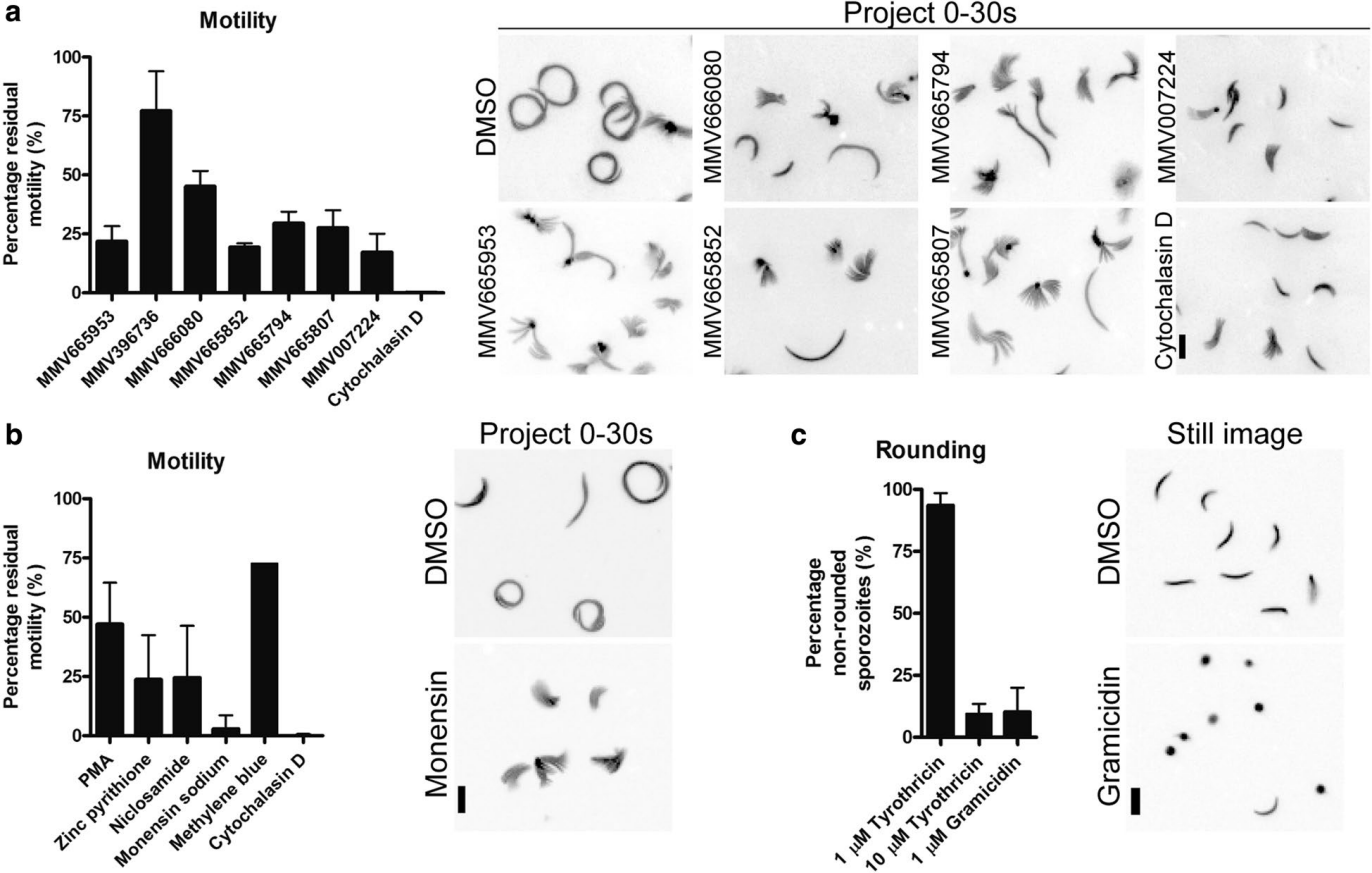
Screening against freshly isolated salivary gland sporozoites. **a** 3 Malaria Box compounds (at 10 µM) displayed potent inhibition of sporozoite motility (> 75% inhibition). **b** Selected drugs from the FDA approved library also displayed potent inhibition at 1 µM, with monensin sodium treatment pronouncedly inhibiting proper sporozoite attachment and motility. In both **a** and **b**, Cytochalasin D was used as a positive inhibitor control. **c** Gramicidin (1 µM) was potent in causing sporozoites to round up, while tyrothricin needed to be at a tenfold higher concentration for a similar effect. Data represented as mean ± SD. Scale bars: 10 µm

The Malaria Box has been used as a screening toolbox for the various stages of the *Plasmodium* life cycle [9] (Fig. 1a). Here, it was investigated whether compounds from this library were capable of directly inhibiting in vitro sporozoite motility. To this end, sporozoites were isolated from freshly dissected mosquito salivary glands and incubated with library compounds for 30 min. The effects of these compounds were then assessed by direct visualization (Fig. 1b). This identified six compounds that reproducibly displayed > 50% inhibition of sporozoite motility (Fig. 1c, Additional file 1: Table S1, Additional file 2: Figure S1). As a positive inhibitor control, sporozoites were treated with 1 µM cytochalasin D (Cyto D), an actin toxin previously shown to be a potent inhibitor for sporozoite movement [52]. Treatment with Cyto D completely abrogated sporozoite motility and resulted in more adherent sporozoites consistent with previous observations (Fig. 2a) [52, 53]. Interestingly, of the hits identified, there were two pairs that shared the same starting scaffold (MMV665953 and MMV665852; MMV665794 and MMV007224). MMV665953 and MMV665852 are part of a family of four related compounds in the Malaria Box with the other two compounds (MMV000911 and MMV001318) not showing any noticeable activity against sporozoites in the pilot screen. In terms of potency, three compounds (MMV665953, MMV665852 and MMV007224) displayed approximately > 75% inhibition (Fig. 1a). However, due to reported toxic effects on hepatocytes [9] and a lack of a currently available topical treatment, these compounds were not further tested in vivo.

### A sub-set of compounds from an FDA-approved drug library are micromolar inhibitors of sporozoites

A previous study made use of an FDA-approved drug library to screen for liver stage drug candidates, resulting in the identification of decoquinate as a promising multistage anti-malarial [8]. In this study, sporozoites were incubated with the compounds and added to liver cells. As a read-out, the development of liver stage parasites was used. This could be affected by either inhibiting liver stage growth or by liver cell invasion or gliding motility of sporozoites. A pilot screen was performed on the 97 drugs that were anticipated to have an effect on extracellular sporozoite motility and possibly not on liver development (Additional file 1: Table S1). From these only a small set of compounds showed prominent inhibition at the screening concentration of 1 µM (> 75% inhibition compared to DMSO control, Fig. 2b, c). These included zinc pyrithione, niclosamide and ionophores monensin sodium and gramicidin. Interestingly, monensin sodium completely blocked sporozoite motility by affecting proper sporozoite adhesion to the glass surface while not affecting shape (Fig. 2b). Gramicidin S treatment resulted in a pronounced rounding up of sporozoites presumably through membrane destabilization (Fig. 2c). Curiously tyrothricin, a mixture of tyrocidines and gramicidins, had a lesser effect on sporozoite morphology than gramicidin alone. Nonetheless at 10 µM concentration a similar effect on sporozoites could be observed as for 1 µM gramicidin. Given this observation, and that an inexpensive topical gel formulation containing tyrothricin (and hence gramicidin) was available, the antibacterial Tyrosur^®^ gel was selected for further in vivo analysis.

### Pre-treatment with Tyrosur^®^ gel does not significantly affect sporozoite infection ability in mice

**Fig. 3:**
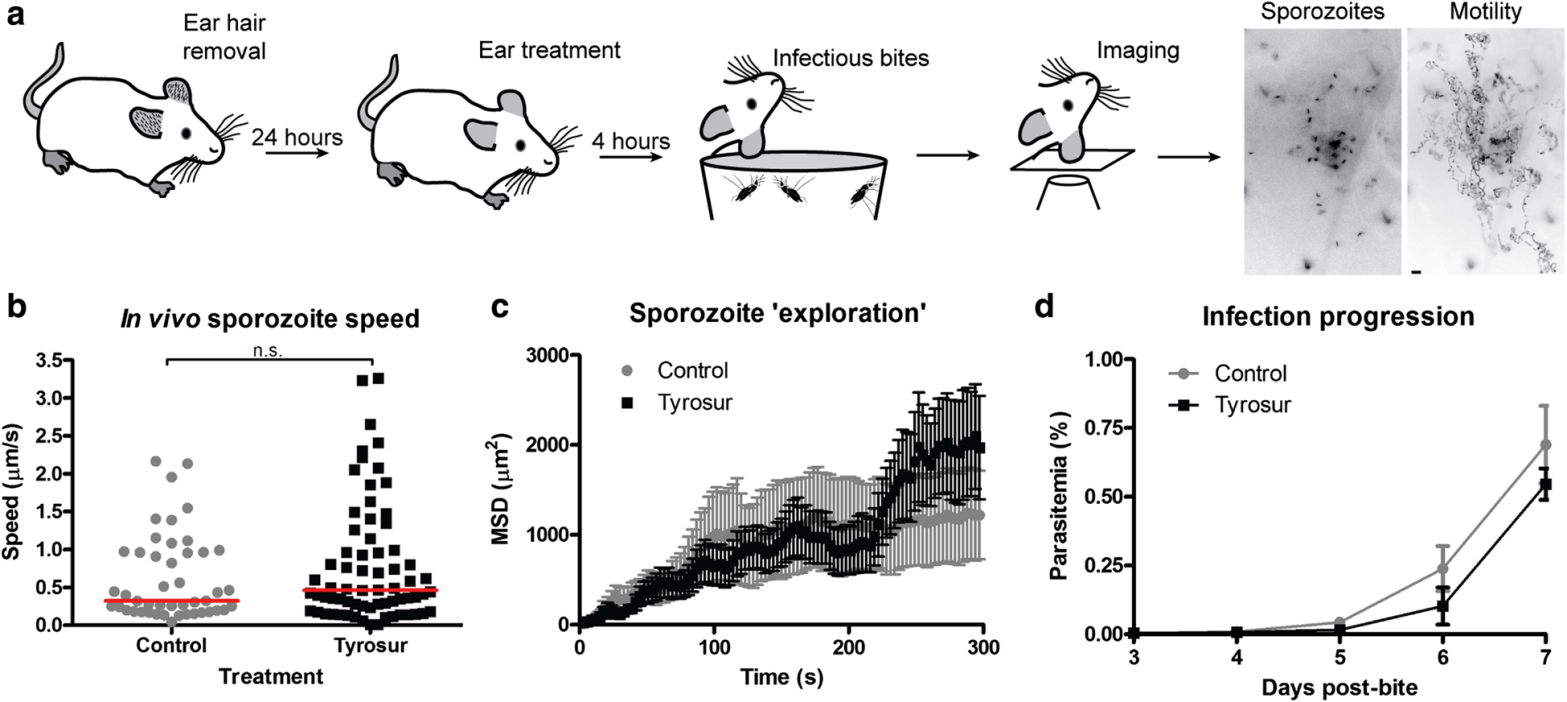
Pre-treatment with a tyrothricin and gramicidin containing topical gel (Tyrosur^®^) did not inhibit in vivo parasite progression. **a** Mice ear hair was removed 24 h before the experiment and mosquito bites 4 h post-topical treatment. Anesthetized mice were transferred to a heated chamber and the bite site imaged to reveals sporozoites (left image). Time lapse recording enabled analysis of migration as shown exemplary by a projection of the sporozoite path over 450 s (right). Scale bar: 10 μm. **b** In vivo imaging of sporozoites deposited in the skin by mosquito bites moved at similar speeds (Mann–Whitney test, red line indicates median speed); and, **c** with similar mean square displacement (MSD) when compared to controls. **d** Mice were monitored after mosquito bite to assess any ‘post-skin’ effects of Tyrosur treatment. Parasite emergence in the blood was similar between groups indicating no prominent effect of Tyrosur after skin exit. Data represented as mean ± SEM

Tyrosur^®^ gel is used to treat and prevent infections of the skin [54, 55]. In order to assess whether pre-treatment of mouse skin with Tyrosur^®^ gel was able to affect the ability of sporozoites to infect new hosts, a mouse model was employed (Fig. 3a). Mice were treated with Tyrosur^®^ or control gels 4 h prior to exposure to infected mosquitoes that were allowed to probe and feed on the treated (or control) region. Mice were subsequently placed on a heated stage of a microscope and fluorescent sporozoites filmed as they migrated in the skin (Fig. 3a). Image analysis revealed no difference between the two groups of mice in terms of sporozoite speed and migration pattern (Fig. 3b, c). Thus, pre-treatment of mice ears with a topical application of gel did not affect sporozoite motility in the host skin. Consistent with these observations, mice exposed to infected mosquito bites showed no significant difference in prepatent period compared to controls, suggesting that this treatment also does not significantly affect sporozoite viability after skin exit and entry into the liver (Fig. 2d).

## Discussion

The skin phase of *Plasmodium* is surprisingly often overlooked in reviews describing drug discovery or vaccination strategies in the malaria field [28]. Not only is this unfortunate in terms of omission of an important part of the parasite biology, but it also misses the important consideration of the skin being an additional area for infection prevention. The possibility of skin phase prophylaxis has been proposed in previous studies [29–32]. Indeed, antibodies to the major surface protein of sporozoites have been shown to affect sporozoite movement in the skin [42] and could therefore contribute to the effects of the RTS,S vaccine [56, 57]. Many groups have performed screens assessing for antimalarial candidates acting at various or combined stages of the parasite life cycle [8–27]. However, to date only one paper has specifically screened and analysed inhibitors of whole sporozoites that could in principle directly act on motile parasites in the skin itself, although it investigated sporozoite-liver cell interactions [32]. A second paper made use of a computational screen for a particular complex of proteins and evaluated the hits on sporozoites yet did not screen libraries on whole sporozoites [58].

In this study, two compound libraries were screened to test for in vitro sporozoite inhibition. The MMV Malaria Box contained three molecules that fulfilled the criteria of potent inhibition of motility (MMV665953, MMV665852, MMV007224). These were part of the list also identified in two separate liver stage screens (which identified 43 candidates) but it is important to note that the compounds identified in the current study were all previously identified as toxic for hepatocytes [9]. Nonetheless, the shorter list of identified compounds might still serve as useful leads in stopping sporozoites that now require further optimization for delivery. Further, these hits also assist the understanding of the liver stage hits identified previously. These data strongly suggest that the other liver stage hits observed for Malaria Box compounds published previously [9] are probably acting either during invasion of the hepatocyte and/or during subsequent intracellular development, but not before. The small number of hits that were identified as sporozoite inhibitors from the blood stage inhibiting Malaria Box library also suggest that the sporozoites show few overlapping targets with other stages. Indeed there is strong evidence for global downregulation of translation in sporozoites [59, 60] and thus fewer targets could be available.

MMV665953 and MMV665852 were potent molecules while structurally similar compounds of the *N*,*N*′-Diarylurea backbone family (MMV000911 and MMV001318) were not. The consistent feature of the two hits is the preservation of the *meta/para* halides and thus these functional groups should be considered in any subsequent derivative design. The current suggested target for this group of compounds is possibly cGMP-dependent protein kinase (PKG) [9], a key regulator of multiple cellular processes. Interestingly, PKG has been shown to be involved in phosphorylation of gliding machinery components and is important for both merozoite red blood cell invasion and ookinete motility [61–63]. It is thus reasonable that inhibition of this kinase would affect sporozoite motility as well, although inducible knockout of PKG did not appear to affect liver cell invasion of sporozoites suggesting additional targets of these compounds and/or compensation by other kinases after deletion [64].

Thrombospondin-related anonymous protein (TRAP) is a prominent parasite adhesin involved in parasite gliding and organ penetration [34, 65, 66]. The other potent hit from the MMV library, MMV007224, has been recently identified as a molecule that might immobilize an aldolase–TRAP interaction thereby affecting sporozoite motility [58]. While aldolase does not have a direct role in motility [67, 68], affecting TRAP dynamics on the parasite plasma membrane by stabilizing a non-specific interaction could have consequences for efficient motility. The aldolase–TRAP study also identified other compounds from the library that were not notably active in the assay used here, presumably because these displayed weaker activities compared to MMV007224. Taken together, this shortlist of MMV malaria box compounds are useful in understanding contributors to parasite motility and could be used as leads to generate selective agents that stop sporozoite motility prior to invading the liver.

In the second screen, 97 compounds were used from a study that screened for liver stage inhibitors [8]. In that study, sporozoites were added together with the compounds to hepatocytes and the read out was liver stage growth. Thus, this assay setup could not distinguish between a compound affecting parasite motility, invasion, viability or growth. Four potent molecules inhibited sporozoite motility at 1 µM concentration. Of these four compounds the two most potent candidates (monensin and gramicidin) belonged to the ionophore class of compounds. This category of antibiotic is particularly attractive since it targets membranes and therefore has reduced likelihood of generation of resistant strains [69]. Monensin is a polyether antibiotic that, when inserting into membranes, results in sodium and potassium fluxes that negatively affects ion homeostasis within cells [70]. It has been employed in ruminant cattle, primarily for the treatment of coccidiosis. Monensin has been shown to be active against *Plasmodium* at different stages including asexual blood stages, gametocytes and oocyst formation [71, 72] although long incubation times were required. Interestingly, pretreatment of hepatocytes with monensin had a potent effect on sporozoite and *Toxoplasma* tachyzoite invasion; while pre-treatment of sporozoites with 1 µM monensin before applying to the hepatocytes only resulted in a 70% reduction [70]. This suggested the primary potency was mediated through host cell mediated effects. At the same concentration in the assays used in this study, there was a reduction in sporozoite motility. This difference is probably best explained by the different environments of sporozoite motility. It is reasonable to suggest that effects in a simple 2D environment (our assay) has more striking effects on adhesion while the three-dimensional environment during cell traversal [70] could require more compound for the same effect. Indeed a similar effect between environments has been observed with mutated parasite lines, which were largely unable to move in 2D but showed no defect in 3D [73, 74]. Surrogate systems that mimic the three dimensional nature of the skin are currently being developed that could be used to test any future potential drug candidates in a 3D environment prior to in vivo testing [75].

Tyrothricin is isolated from *Bacillus brevis* and consists of a mixture of cyclic decapeptides, gramicidin S and tyrocidine A. Similar to monensin, gramicidin is a polypeptide that produces membrane pores and affects cation gradients [76]. Given the general targeting of membranes, it is not surprising that ionophores have been shown to be effective in inhibiting *Plasmodium* blood stage growth [71, 77–79]. It is interesting that tyrothricin, a mixture of ionophoric peptides, was not as potent in sporozoite rounding as gramicidin. This suggests that the active ingredient against sporozoites is more specifically gramicidin (which is only a fraction of tyrothricin). Tyrocidine peptides appear to be the primarily potent molecules against blood stage parasites with an IC50 value in the low micromolar range [79]. The difference between potencies of tyrothricin and gramicidin against sporozoites might provide a subtle hint that membrane susceptibilities are different across the life cycle. Different effects between other ionophores were also noticed: monensin affected proper sporozoite adhesion while gramicidin led to rounding up of sporozoites. While the molecular details are currently not clear regarding the altered response, it is reasonable to speculate that differences in pore sizes formed by these agents could affect dynamics of both the plasma and organellar membranes [80–84].

Ionophores have been used effectively in both the topical and systemic treatment of gram-positive bacterial infections [85]. Given the micromolar in vitro inhibition of the tyrothricin mixture, with the presence of gramicidin in its overall composition and the availability of a gel formulation for topical application, we decided to further analyse Tyrosur^®^ gel for possible in vivo effects on deposited sporozoites. However, treatment of mice ears with Tyrosur^®^ gel did not significantly affect sporozoite infection ability after mosquito bite. Given the lack of inhibition even with large amounts of gel applied, it does suggest that the drug is not able to sufficiently permeate the dermal layer where sporozoites are predominantly located after the bite. Thus, reduced drug accessibility to the essential skin layer could be the major cause of treatment failure. Recent publications indicate good progress on developing and modelling dermal penetration of exogenous molecules [86–89] but there still remains a long way to go before this could be used effectively in the field. Since this is a critical point, future work should thus focus on applying enhanced delivery methods that might allow targeting of the parasite and prevention of infection. Further, orally administered drugs should not be excluded as it is also possible that, if able to permeate the dermal layer via the blood, one could achieve the same effect of sporozoite motility in the skin. Such dermal penetration has been suggested previously in the case of passive intravenous transfer of CSP antibodies [42]. Our platform could thus also be used to quantitatively evaluate other antibodies against sporozoite proteins.

## Conclusion

In this study, a screening pipeline has been established and utilised to directly assess the potential effects of different drug candidates on extracellular sporozoite motility. Through this approach, a small set of molecules have been identified, including three MMV Malaria Box compounds and two ionophores, that have potent effects on sporozoites viability in vitro. However, much further development is needed such that compounds can be effectively delivered to an intact dermis. Given the small numbers of hits on sporozoites, screening of larger libraries is needed.

## Additional files

**Additional file 1: Table S1.** Initial pilot screen of MMV Malaria Box and FDA approved compounds.

**Additional file 2: Figure S1.** Structures of inhibitors showing >50% inhibition.
