# Supplementary figures and images for: Screening for potential prophylactics targeting sporozoite motility through the skin
Source: Malar J. 2018 Aug 31;17:319. doi: 10.1186/s12936-018-2469-0 (PMC6119338; doi:10.1186/s12936-018-2469-0)

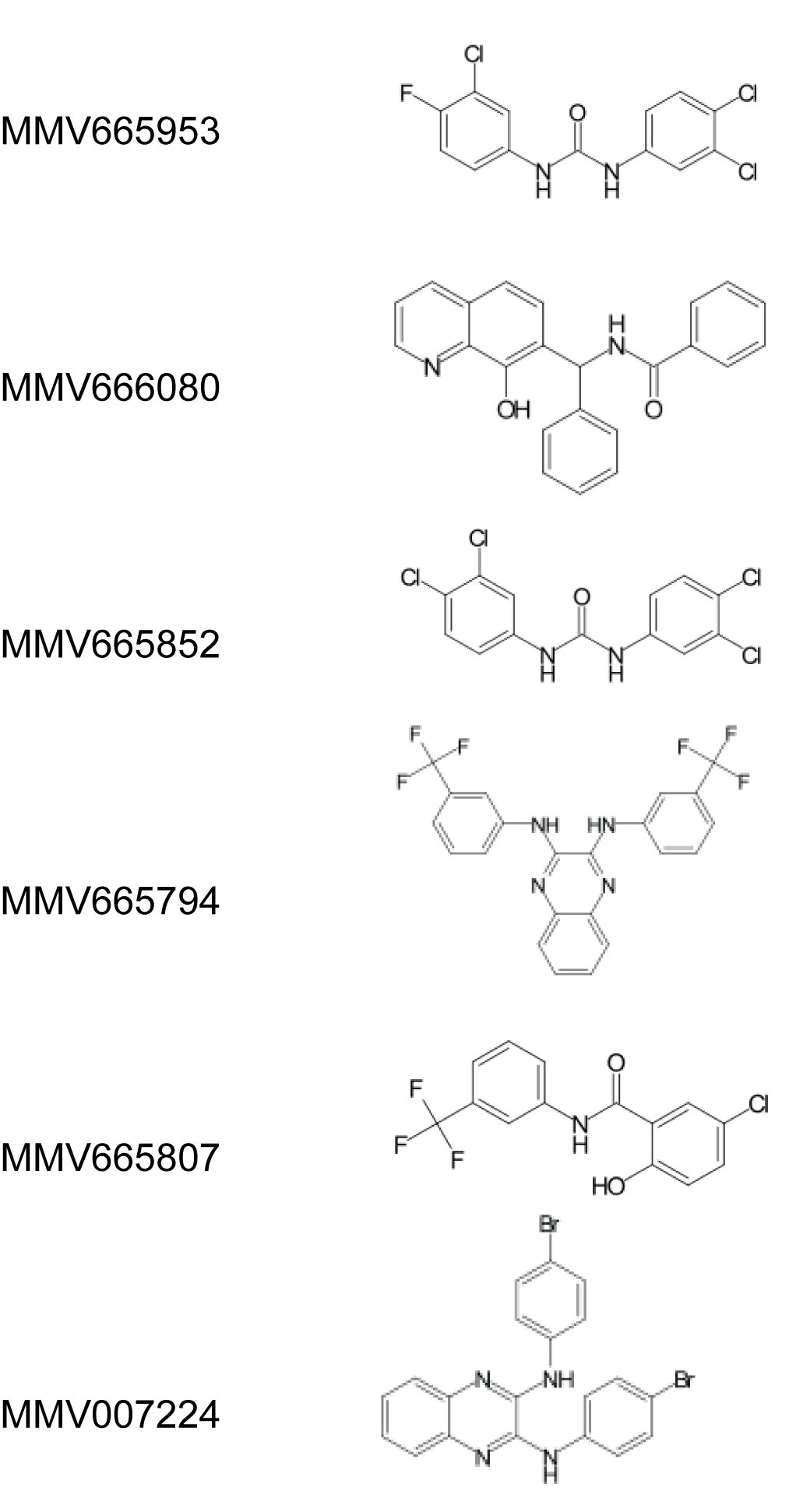

Supplement: Supplementary file 2 — Additional file 2: Figure S1. Structures of inhibitors showing >50% inhibition. [file 12936_2018_2469_MOESM2_ESM.tif]
